# Zebrafish Models of Cancer Therapy-Induced Cardiovascular Toxicity

**DOI:** 10.3390/jcdd8020008

**Published:** 2021-01-22

**Authors:** Sarah Lane, Luis Alberto More, Aarti Asnani

**Affiliations:** 1 CardioVascular Institute, Beth Israel Deaconess Medical Center, Boston, MA 02215, USA; slane3@bidmc.harvard.edu (S.L.); lmorever@bidmc.harvard.edu (L.A.M.); 2 Harvard Medical School, Boston, MA 02115, USA

**Keywords:** cancer therapy, cardiovascular toxicity, zebrafish, cardiac development, vascular development

## Abstract

Purpose of review: Both traditional and novel cancer therapies can cause cardiovascular toxicity in patients. In vivo models integrating both cardiovascular and cancer phenotypes allow for the study of on- and off-target mechanisms of toxicity arising from these agents. The zebrafish is the optimal whole organism model to screen for cardiotoxicity in a high throughput manner, while simultaneously assessing the role of cardiotoxicity pathways on the cancer therapy’s antitumor effect. Here we highlight established zebrafish models of human cardiovascular disease and cancer, the unique advantages of zebrafish to study mechanisms of cancer therapy-associated cardiovascular toxicity, and finally, important limitations to consider when using the zebrafish to study toxicity. Recent findings: Cancer therapy-associated cardiovascular toxicities range from cardiomyopathy with traditional agents to arrhythmias and thrombotic complications associated with newer targeted therapies. The zebrafish can be used to identify novel therapeutic strategies that selectively protect the heart from cancer therapy without affecting antitumor activity. Advances in genome editing technology have enabled the creation of several transgenic zebrafish lines valuable to the study of cardiovascular and cancer pathophysiology. Summary: The high degree of genetic conservation between zebrafish and humans, as well as the ability to recapitulate cardiotoxic phenotypes observed in patients with cancer, make the zebrafish an effective model to study cancer therapy-associated cardiovascular toxicity. Though this model provides several key benefits over existing in vitro and in vivo models, limitations of the zebrafish model include the early developmental stage required for most high-throughput applications.

## 1. Introduction

Cardiovascular disease and cancer are leading causes of morbidity and mortality worldwide. The presence of pre-existing cardiovascular disease not only affects clinical outcomes in patients with cancer, but there is also increasing evidence to support a causal relationship between cancer and cardiovascular disease. For instance, neurohormonal activation in heart failure or inflammation and oxidative stress in atherosclerosis are associated with a higher incidence of cancer in this population with cardiovascular disease [1,2].

Even though modern cancer treatment strategies have led to higher cancer survivorship rates, many regimens used currently are associated with cancer therapy-induced cardiovascular toxicity [3]. For instance, in a large cohort of patients treated with anthracyclines, the incidence of cardiac dysfunction was 9% within the first year after completion of treatment [4], with higher rates of cardiomyopathy reported in patients with pre-existing cardiovascular disease. Survivors of childhood cancer have an 8-fold higher cardiac death rate than the age-matched and sex-matched national average [5]. Targeted cancer therapies affecting the VEGF signaling pathway can lead to a broad range of cardiovascular complications, including hypertension, cardiomyopathy, arrhythmia, pericardial effusion, and QT prolongation.

For most cancer therapeutics, the underlying molecular mechanisms driving cardiovascular toxicity are not fully understood despite decades of research, such as with the anthracyclines. Importantly, for most cardiovascular toxicities, it is not understood whether these represent on-target or off-target effects. A more comprehensive understanding of the molecular mechanisms would enable the development of effective cardioprotective strategies and improve quality of life and survivorship in patients with cancer [6].

The zebrafish has emerged as a high throughput animal model to tackle these mechanistic questions and to identify novel cardioprotective strategies [7,8]. Zebrafish embryos are small and can be easily visualized in 96-well plates, requiring only a few hundred microliters of embryo medium to survive. As such, chemical screening can be performed quickly with a large number of small molecules to assess dose-response relationships. Zebrafish embryos are optically transparent, have high fecundity, and considerable genetic similarity with humans [9]. Cardiogenesis pathways in humans and zebrafish share many similarities, and several models for both cardiovascular diseases and cancer have previously been developed in the zebrafish. The ability to use zebrafish to study both cardiovascular diseases and cancer, along with the advantages of performing high throughput discovery studies, makes the zebrafish a desirable model for cancer therapy-associated cardiotoxicity. Furthermore, models that recapitulate cancer therapy-associated cardiovascular toxicity have successfully been developed and have the potential to provide new insights into fundamental cardiovascular biology outside the context of cancer. This review addresses zebrafish as a model for cancer therapy-induced cardiovascular toxicity, emphasizing the role of zebrafish in studying both cardiovascular diseases and cancer biology for a comprehensive assessment of chemotherapy-induced cardiotoxicity.

## 2. Zebrafish as a Model for Cardiovascular Disease

**Figure 1:**
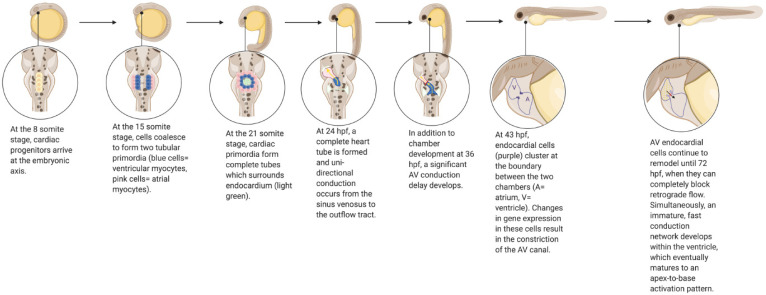
Development of the Zebrafish Cardiovascular System. Created with BioRender.com.

Zebrafish have long been used as a model to study molecular pathways in developmental biology, particularly as they apply to human cardiovascular disease [10]. As in humans, the zebrafish heart develops from a heart tube derived from cardiac progenitors [11] (Figure 1). Unidirectional electrical conduction begins at 24 h post-fertilization (hpf), and the heart tube begins to loop shortly thereafter at 30 hpf, forming distinct chambers [11,12]. Atrioventricular valve formation occurs around 43 hpf, and continues to develop until 72 hpf, at which point valve leaflets are able to completely block retrograde flow [13,14,15]. From this point onwards (72–96 hpf), an immature fast conduction network develops within the ventricle, which eventually fully matures to an apex-to-base activation pattern when the ventricular apex has formed [12]. Cardiogenesis in mammals and zebrafish requires many of the same essential genes and regulatory networks; however, the zebrafish requires much less time than humans to complete these events [10]. Though there are clear structural differences between the four-chamber human heart and the two-chamber zebrafish heart, a high degree of genetic orthology between the two species, combined with high fecundity and easy visualization of phenotypes, render the zebrafish a desirable model for studying a wide variety of cardiovascular processes and pathologies.

Despite anatomical differences between human and zebrafish hearts, the two species share several key electrophysiological characteristics that are challenging to recapitulate using existing rodent models. Zebrafish action potentials have all of the same phases of the human action potential, with the exception of rapid phase-1 repolarization. Additionally, the resting heart rate of a zebrafish (about 150 beats/min in adult fish) is significantly closer to the average human heart rate than that of a mouse, which is typically around 600 beats/minute [16]. Both zebrafish and human action potentials have a long plateau phase which produces a distinct QT-interval on an ECG; mice lack this long plateau phase [17]. This characteristic of the zebrafish model is particularly useful for studying cancer therapy associated cardiotoxicity; several classes of targeted therapies, including VEGF pathway inhibitors, Bcr/Abl kinase inhibitors, and histone deacetylase inhibitors, have been documented to cause prolongation of ventricular depolarization, as measured by the corrected QT interval (QTc), in patients with cancer [18]. Zebrafish have previously been used as a model to study drug-associated QT prolongation, which can cause fatal arrhythmias. For instance, zebrafish can be used to study QT prolongation associated with various drugs by anesthetizing adult fish and then placing them on a damp sponge, or by oral administration with a perfusion needle [16]. QT prolongation has also been studied in the zebrafish model using genetically modified fish lines, including transgenic lines with mutant *kcnh2* alleles to study and screen for therapeutics for Long QT Syndrome Type II [19,20,21,22,23,24]. Similar heart rates and analogous ECG parameters for atrial, atrioventricular, and ventricular depolarization are key advantages of the zebrafish model over murine models for studying human electrophysiology. While zebrafish and humans share many key similarities in electrophysiology, differences exist in ion channel molecular composition and functioning, such as significant sensitivity differences of inward rectifying channel Kir2 and non-homologous origins of delayed rectifier K+ currents, that may limit the use of this model for certain human electrophysiological conditions [17].

Similarly, several zebrafish models of heart failure have been developed. Heart failure can arise from coronary artery disease, hypertension, ischemia, myocardial infarction, and inherited and acquired cardiomyopathies. Several cancer therapies, including anthracyclines and targeted therapies such as VEGF pathway inhibitors, are associated with an increased risk of developing cardiomyopathy. Ex vivo models of cardiomyopathy are limited by the availability of both healthy and diseased human cardiac tissue, and tissue models fail to accurately recreate the complex, multi-tissue phenotypes of heart failure. Several mammalian models for heart failure exist; however, using such models for drug discovery and mechanistic studies can be costly and time consuming, prompting the need for an affordable, high throughput model such as the zebrafish. Unlike humans, embryonic zebrafish are not dependent on a functional circulatory system for survival until 7 dpf. In the absence of a fully developed circulatory system, embryonic zebrafish cells are oxygenated through passive diffusion. This unique characteristic of zebrafish development provides an opportunity to study genetic mutations or cardiomyopathies that severely compromise the cardiovascular system, which can be difficult to study in mammalians [25]. Transgenic zebrafish lines have previously been used to study a lethal mutation that causes dilated cardiomyopathy [26,27], and another which produces a deficit in cardiac troponin T, a mutation in which is responsible for 15% of familiar hypertrophic cardiomyopathy in humans and leads to an increased risk of early death [28]. In addition to studying the potential genetic bases of heart failure, the zebrafish is emerging as a robust screening platform for potential human heart failure therapies. Transgenic reporter zebrafish lines, including a line that expresses Firefly luciferase downstream of the promoters for *nppa* and *nppb* genes, have been validated as a screening tool for modifiers of cardiac natriuretic peptide expression with several established cardioactive agents [29]. The ability to recapitulate a heart failure phenotype in the zebrafish model, and successfully induce known biomarkers of cardiac injury in response to cardioactive agents, provides an exciting opportunity for researchers to screen for and study heart failure therapeutics in a cost-effective, high throughput manner.

Finally, the zebrafish offers a unique opportunity to study angiogenesis. There are several human diseases caused by or associated with the dysregulation or abnormal growth of blood vessels, prompting the need for an effective model to study these processes. In humans, inadequate vessel growth can lead to ischemia, whereas unregulated growth can promote cancer or inflammatory disorders. Additionally, cancer can cause unregulated vessel growth by modulating angiogenesis and vasculogenesis, aiding in the proliferation and metastatic spread of cancerous cells. Several cancer therapies, such as the VEGF pathway inhibitors sorafenib and sunitinib, are effective at targeting these processes in cancerous tissue, but also disrupt normal angiogenic processes and are associated with cardiovascular toxicity in patients [30,31,32]. There are several existing in vitro models for angiogenesis, such as proliferation assays, tubule formation assays, and organ explant assays [33,34,35,36,37,38,39]. However, all lack the ability to model the interactions between supporting cell types, and the overall complexity of a whole organism. Numerous in vivo models have been developed as well, such chorioallantoic membrane assays, hind limb ischemia, and the Matrigel plug assay. These models have been used primarily to assess outcomes of a specific procedure or treatment [40,41,42,43]. Zebrafish have a basic vasculature plan similar to that of humans as well as conservation of major angiogenesis modulators, such as the tyrosine kinase domains of vascular endothelial growth factor receptor 2, making this an ideal in vivo model to study vasculogenesis, angiogenesis, and vascular regeneration [44,45,46,47]. There are several publications which describe in detail the molecular basis of angiogenesis and vasculogenesis in zebrafish [48,49]. With the increasing popularity of the zebrafish as a model to study vasculogenesis and angiogenesis, several imaging tools, such as microangiography and time-lapse imaging, have been harnessed to study vascular development in zebrafish embryos [44]. In addition, transgenic lines can be combined to express fluorescent proteins on different cell types [48,50,51], which can be observed simultaneously during angiogenesis, and reporter lines can be used to screen for small molecules that compromise vascular integrity [52,53]. The zebrafish has previously been used to study numerous anti-angiogenic cancer therapies [54,55,56,57] and is particularly well suited to study these types of cancer therapies due to the ability to visualize angiogenesis in real-time, the relative ease of creating transgenic lines for studying tissue-specific gene expression, and the amenability of creating xenograft models of several human cancers.

## 3. Zebrafish as a Model to Study Cancer Biology

With the evolution of genome editing technology, the zebrafish has become an attractive vertebrate model to study cancer biology. In studying cardiovascular toxicity of cancer therapeutics, it is imperative that screening approaches assess the relationship between mechanisms leading to cardiac toxicity and those required for antitumor efficacy. For instance, use of the FDA-approved cardioprotective agent dexrazoxane in patients treated with anthracyclines is limited by concerns that this agent may interfere with the antitumor effect and induce secondary malignancies, highlighting the importance of studying cardiovascular and cancer phenotypes in parallel. The relative ease of genetic manipulation of zebrafish at early embryonic stages by microinjection of oncogenes or knockout of tumor suppressors facilitates assessment of tumor behaviors in vivo. The creation of transgenic zebrafish cancer models began in 2003 with injection of the *c-myc* transcription factor in zebrafish embryos at the one-cell stage of development under control of the *Rag2* promoter, driving expression of this oncogene in lymphoid cells. Five percent of injected embryos ultimately developed T cell acute lymphoblastic leukemia, as assessed by visualization of leukemic cells expressing *MYC* fused to a green fluorescent protein (GFP) [58].

Early transgenic zebrafish cancer models were developed by co-injection of a *Tol2* transposase messenger RNA (mRNA) with a plasmid DNA vector consisting of *Tol2* sites, a human oncogene, a tissue specific promoter, and a marker, such as a fluorescent protein. An important limitation of this model is the limited number of transgenes generated per line, in contrast to the complex genetic interactions between oncogenes and tumor suppressor genes that occur in human cancer biology. The CRISPR-Cas9 gene-editing technique has partially overcome this issue by creating tumor suppressor knockouts through the injection of guide RNAs (gRNAs) carried by a plasmid vector containing a Cas9 enzyme [59,60]. The ability to inject multiple gRNAs targeting different genes in a multiplex fashion aims to simulate the complexity of human cancer genotypes in in vitro and in vivo models. For instance, Ablain et al. developed a zebrafish model of human melanoma expressing the oncogenes BRAF-V600E, KIT-K642E, and NRAS-Q61R, with inactivation of the *tp53* tumor suppressor gene in a melanocyte-specific manner [59].

Furthermore, the zebrafish is an ideal transplant recipient for cancer cells. Some zebrafish lines, such as the *casper* strain, have translucent skin that facilitates tracking of injected tumors, such as human leukemia cells, in both larvae and adults [61]. The ability to inject cancer cells in hundreds of larvae or dozens of adult zebrafish in a single day, and the relatively low cost for husbandry and maintenance of these lines compared to mice, make them even more appealing for cancer xenografts. As the adaptive immune system becomes functionally mature around 1 to 2 weeks post-fertilization, injection of allogeneic cancer cells can be performed in larvae in the absence of an immunocompromised animal model. However, important limitations of the larval xenotransplantation model include the number of tumor cells that can be injected, and the survival and proliferation of these cells until they are rejected [62,63]. An adult immunodeficient zebrafish line lacking T and B cells (*prkdc* -/- and *il2rga* -/-) was optimized for tumor cell proliferation and long-term engraftment. An advantage of the adult zebrafish xenotransplantation model is the higher number of cells that can be injected per recipient, as well as the increased longevity whereby fish can live at 37 °C for months, resembling human conditions for tumor growth [63,64].

Cancer cell differentiation, proliferation, and migration in transgenic zebrafish models and zebrafish cancer xenografts provide an excellent opportunity to study tumor biology under similar circumstances as in human cancer biology. These models, combined with the ease of using zebrafish for high throughput chemical screens, can enable the identification of novel anti-proliferative agents. A wide array of cancer therapeutics can be tested in zebrafish, ranging from cytotoxic drugs such as doxorubicin to targeted cancer therapies. Interestingly, the antitumor effect of these anti-proliferative agents can be evaluated alongside any cardiac toxicity secondary to cancer therapy. In one study, zebrafish and mice xenograft models were injected with A549 cells and then used to assess co-treatment of doxorubicin with digoxin, an inotropic agent used in heart failure [65]. Co-treatment with digoxin enhanced the antitumor effect of doxorubicin in both organisms and reduced cardiotoxicity caused by doxorubicin in mice, as quantified by the heart size and cardiomyocyte size postmortem. Similarly, a zebrafish model of human cancer was constructed injecting HCT115-GFP cells into the yolk sac of zebrafish embryos 48 h post-fertilization [66]. Twenty-four hours after xenotransplantation, zebrafish larvae were treated with doxorubicin by retro-orbital injection of this drug via the eye socket. Cardiac function was evaluated within 48 h post-treatment by 3D cardiac imaging with a confocal microscope equipped with a rapid resonant scanner, and changes in tumor size were assessed by 3D fluorescence. Heart rate, end-diastolic volume, end-systolic volume, and ejection fraction were included as part of the cardiac assessment.

Zebrafish cancer avatars provide unique advantages to study cancer biology. The low cost, large scale, and short time to develop a tumor model make zebrafish suitable for this purpose. The time to develop patient-derived xenografts (PDXs) in zebrafish larvae and adults is 5 to 7 days and weeks to months, respectively. Development of PDXs in mice takes weeks to months at a high cost and low drug screening throughput. With advances in precision medicine, development of PDXs in zebrafish has the potential to inform clinical decisions on an individualized level [67]. For instance, zebrafish PDXs have been used to assess the effectiveness of antiproliferative agents on primary tumor biopsies in order to personalize cancer therapy [68]. In the preclinical setting, zebrafish tumor models can lend insight into the potential for cardiovascular toxicity with new anti-proliferative agents, with the possibility of evaluating cardiac function simultaneously.

## 4. Zebrafish as a Model for Cancer Therapy-Associated Cardiovascular Toxicity

Given the range of zebrafish models available to study both cardiovascular and cancer biology, zebrafish can serve as an important tool to screen new cancer therapeutics for potential cardiovascular toxicity, and importantly, to interrogate the on- and off-target molecular mechanisms leading to cardiovascular toxicity. The zebrafish model also serves as an optimal platform to identify novel cardioprotective agents. In early developmental stages, many small molecules can be administered simply by dissolving them in embryo medium or, for lipophilic agents, by injection into the yolk sac. For adult zebrafish, oral gavage and intraperitoneal injection can be performed [69]. The optical transparence of zebrafish embryos and the use of translucent strains such as *casper* allow for visualization of cardiac, vascular, and tumor phenotypes simultaneously through either bright-field or fluorescent microscopy.

### 4.1. Limitations of Cell Culture and Mammalian Models of Cancer Therapy-Associated Cardiotoxicity

Although several in vitro and in vivo models have been established to study cancer therapy-associated cardiovascular toxicity, each has limitations that may hinder the translation of preclinical observations to human pathophysiology. Human induced pluripotent stem cells (hiPSCs) have emerged as an important tool to identify patients who may develop cardiovascular toxicity after treatment with specific cancer therapies. For instance, hiPSC-CMs isolated from patients treated with doxorubicin who developed cardiovascular toxicity were compared to those from patients treated with doxorubicin who maintained preserved cardiac function. hiPSC-CMs isolated from patients with cardiovascular toxicity demonstrated evidence of decreased mitochondrial function, impaired calcium handling, increased reactive oxygen species production, and decreased antioxidant pathway activity [70]. Although hiPSC-CMs have the advantage of reflecting molecular pathways directly relevant to humans, as well as the genetic variation seen in patients, there are limitations to this model in the context of cardiovascular toxicity screening. As with other in vitro models, exposure to hemodynamic or neurohumoral influences on the cardiovascular system is typically lacking, and it can be challenging to phenocopy maladaptive cardiovascular responses to hypertension and other cardiac stressors [71].

Rodents provide an in vivo model with cardiac anatomy closely resembling that of the human heart, but fundamental differences in cardiac function, such as markedly higher resting heart rates, result in differences in calcium handling and electrophysiology relative to human cardiomyocytes. Rodents have been successfully used to study anthracycline-associated cardiotoxicity, but similar experiments with tyrosine kinase inhibitors have been less consistent with observed toxicities in patients. For instance, rodents treated with VEGF pathway inhibitor sunitinib, which is associated with cardiomyopathy in humans, are able to maintain left ventricular function even when tested alongside additional stressors like moderate hypertension [54]. Larger mammalian models like the pig provide the advantage of cardiac function that closely resembles that of humans, but their large size markedly increases the cost of studies and can raise ethical concerns, limiting the use of pigs as a high- or even medium-throughput model. Though mammalian models and in vitro iPSC models provide unique benefits for studying cancer therapy-associated cardiovascular toxicity, an in vivo model such as the zebrafish that provides the complexity of a whole organism with a high degree of homology in cardiovascular function can provide important complementary information to these models when investigating mechanisms of cardiovascular toxicity.

### 4.2. Benefits of Zebrafish as a Model for Cancer Therapy-Associated Cardiovascular Toxicity

The zebrafish has been successfully used as a model for several cancer therapy-associated cardiotoxicities that are commonly observed in human patients. Acute doxorubicin toxicity has been particularly well studied in zebrafish, whereby a dose of doxorubicin 100 μM added to the embryo medium results in pericardial edema, decreased blood flow through the tail vasculature, and decreased cardiac contractility that can be visualized using a standard light microscope [7]. Our laboratory used this model to identify Cytochrome P450 Family 1 enzymes as an important therapeutic target in anthracycline cardiomyopathy [8,72], not only in acute cardiotoxicity but also in chronic doxorubicin cardiomyopathy models in adult mice. A chronic model of doxorubicin cardiomyopathy has also been developed in adult zebrafish, whereby hearts from fish treated with doxorubicin exhibited myofibril disarray and fetal cardiac gene expression [73]. Additionally, novel delivery methods for doxorubicin have been explored in the zebrafish model, including mixed-micellar and liposomal formulations, both of which resulted in less cardiotoxicity and lower mortality rates than traditional doxorubicin formulations [74,75]. In addition to anthracyclines, zebrafish have also been used to study the cardiotoxic effects of other chemotherapeutic drugs like alkylating agents. In a zebrafish model, embryos treated with cyclophosphamide developed pericardial edema and other circulation defects [76]. These findings are consistent with the cardiotoxicity that is sometimes observed in patients treated with this therapy [77,78].

In addition to conventional chemotherapies, the zebrafish has the potential to serve as a model for cardiotoxic effects of novel cancer therapies, including small molecule targeted therapies and immunotherapies. The similarity between the zebrafish and human kinomes makes the zebrafish an ideal model to study potentially cardiotoxic tyrosine kinase inhibitors that have been historically challenging to study in rodent models [54]. Transgenic BNP reporter zebrafish lines have been previously developed to assess cardiovascular toxicity of tyrosine kinase inhibitors approved to treat chronic myelogenous leukemia, such as ponatinib [79]. Similarly, an embryonic zebrafish model identified induction of a Raf-independent ERK pathway as a cardioprotective strategy to protect against sorafenib-associated cardiotoxicity [54]. Additionally, a zebrafish model has previously been used to investigate the possible cardiotoxic side effects of the VEGF inhibitors apatinib and bevacizumab. At 120 hpf, bevacizumab-treated embryos had no obvious side effects from the drug; however, apatinib-treated embryos exhibited numerous side effects, notably pericardial edema and decreased heart rate [80]. These results were consistent with the toxicity observed in patients during previous clinical trials [80].

The transparent nature of zebrafish embryos and advances in imaging technology have made it possible to study the morphological and hemodynamic changes associated with cancer therapy-associated cardiovascular toxicity. There are software programs that measure hemodynamic changes like blood flow, heartbeat, and vessel diameter variations, and by obtaining measurements such as blood cell speed, the flow rate through blood vessels can be used to estimate shear stress on endothelial cells in zebrafish treated with a medication of interest [74,81]. Other software packages are capable of detecting beat frequency arrythmias in both the atrium and ventricle, QT & QTc interval, fibrillation, ejection fraction, cardiac arrest, and other parameters/events in myocardial fluorescent fish lines [81]. Such morphological and hemodynamic changes are more challenging to visualize in rodent models.

Another important feature of the zebrafish model is the amenability of the genome for editing, which enables researchers to create transgenic zebrafish lines with relative ease compared to mammalian models. Genetic knockout models can be created to investigate a cardiotoxic drug’s mechanism of action and target. For instance, a chemical screen in zebrafish enabled the identification of Cyp1 enzymes in anthracycline cardiotoxicity, and mutation of the Cyp1 active site using CRISPR/Cas9 prevented the development of cardiotoxicity in fish [72]. One could envision using zebrafish to study variants associated with cardiotoxicity in human genome-wide association studies. Fluorescently labeled zebrafish lines, such as *Tg(cmlc2:gfp)* representing cardiomyocytes or *Tg(flk1:DsRed)* representing endothelial cells can be harnessed to better visualize the expression of genes associated with cardiovascular toxicity [79]. As mentioned previously, a reporter line that expresses Firefly luciferase downstream of BNP, a marker of heart failure, can be used to test a drug for possible cardiotoxic effects [79]. A fish line with a calcium-sensitive reporter in the myocardium like *Tg(cmlc2:gCaMP)* has been used to analyze changes in cardiac conduction [82], and could potentially be used to study electrophysiological disturbances caused by cardiotoxic drugs.

A unique benefit of the zebrafish model is the opportunity to study vascular regeneration, a process difficult to study in mammals and particularly in humans. Zebrafish are able to regenerate their organs, including their heart and fins, even into adulthood. Adult zebrafish are able to regenerate up to 20% of a ventricle after amputation [83]. Rather than occurring through the generation of new cardiomyocytes, this process begins with existing cardiomyocytes [84]. Heart regeneration is suspected to start with the limited dedifferentiation of the cardiomyocytes neighboring the site of injury. These cardiomyocytes disassemble their sarcomeric structure and detach from one another, a process similar to the one that hibernating myocardium in humans undergoes after cardiac injury [84]. Regulators of cell cycle progression, such as *plk1* and *mps1^4^*, are secreted by cardiomyocytes adjacent to the site of injury and promote the rest of the regenerative process [84]. These findings, which point to cardiovascular regeneration occurring through limited dedifferentiation of cardiomyocytes, offer exciting potential to study how this process may be induced in mammals [84].

### 4.3. Considerations for Using Zebrafish in the Study of Cancer Therapies

There are several important considerations when deciding if and how to use zebrafish models to study cancer therapy-associated cardiovascular toxicity. The first is the type of cancer therapeutic being investigated. As noted above, zebrafish have been successfully used to study anthracycline cardiomyopathy and remain a promising model to study targeted therapy-associated cardiovascular toxicity. However, this model is likely not as effective for investigating mechanisms of autoimmune myocarditis elicited by the immune checkpoint inhibitors, which require adaptive immunity that is lacking in the zebrafish embryo model. Additionally, some monoclonal antibodies may not be well-suited for molecular studies in zebrafish due to a fundamental difference in protein isoforms. For instance, trastuzumab appears to bind exclusively to the human isoform of HER2 and is therefore difficult to study in any non-human model.

A second consideration is the possible dosing route(s) for the agent of interest, which depends heavily on its solubility and lipophilicity. Some small molecules can be directly dissolved in fish water during embryonic development and can easily enter the chorion to be absorbed by embryonic fish. Some agents, such as the cardiotoxic antimetabolite 5-fluorouracil, are not well absorbed and instead can be delivered by microinjection [85]. Lipophilic compounds with Log P values greater than 1 are typically best absorbed by the embryo from the medium, so an alternative dosing route like oral gavage (in adult fish) or injection should be considered for chemicals of interest with Log P values less than 1. Though soaking is a quick and relatively easy model to screen for potentially cardiotoxic agents, there are several limitations to this method. The first is that only compounds that can be readily dissolved in embryo medium can be screened; poorly soluble chemicals will not easily be able to enter the chorion which can lead to decreased drug exposure to the embryo and a false-negative phenotypic score. Mass spectrometry can be used to determine the amount of drug in embryonic zebrafish; however, this process can be time consuming and hinders the ability to screen these chemicals in a high throughput manner [76]. Furthermore, soaking is a fundamentally different route of exposure than those typically used for humans, which may affect translation of observations to the clinical setting [86]. A final limitation of soaking is the challenge in extrapolating the therapeutic window of a drug in embryonic fish to rodents and humans [76].

Finally, the type of cardiac dysfunction under investigation must be considered. Embryonic zebrafish are ideal for studying acute drug-induced cardiotoxicity, while adult zebrafish can be used to model chronic and progressive cardiac pathologies, such as cardiomyopathy and heart failure. The ultimate sequelae of cardiovascular toxicity, such as cardiomyocyte death, may not recapitulate human disease due to the unique ability of zebrafish to regenerate cardiac tissue [86].

## 5. Conclusions

Cancer therapy-associated cardiotoxicity represents an increasingly prevalent and potentially life-threatening complication for patients. Effective models to screen for cardiovascular toxicity, as well as new cardioprotective approaches, are needed to improve long-term cardiac health in this patient population. The zebrafish model provides several key advantages over existing in vitro and in vivo models of cardiotoxicity, namely the relative ease of creating transgenic lines, the ability to observe embryonic cardiovascular phenotypes with light microscopy, and the ease with which small molecules can be delivered in a high throughput manner.
